# External Human–Machine Interfaces for Autonomous Vehicle-to-Pedestrian Communication: A Review of Empirical Work

**DOI:** 10.3389/fpsyg.2019.02757

**Published:** 2019-12-10

**Authors:** Alexandros Rouchitsas, Håkan Alm

**Affiliations:** Humans and Technology Division, Luleå University of Technology, Luleå, Sweden

**Keywords:** traffic interaction, human–vehicle interaction, autonomous vehicles, vehicle-to-pedestrian communication, external human–machine interfaces, vulnerable road users

## Abstract

Interaction between drivers and pedestrians is often facilitated by informal communicative cues, like hand gestures, facial expressions, and eye contact. In the near future, however, when semi- and fully autonomous vehicles are introduced into the traffic system, drivers will gradually assume the role of mere passengers, who are casually engaged in non-driving-related activities and, therefore, unavailable to participate in traffic interaction. In this novel traffic environment, advanced communication interfaces will need to be developed that inform pedestrians of the current state and future behavior of an autonomous vehicle, in order to maximize safety and efficiency for all road users. The aim of the present review is to provide a comprehensive account of empirical work in the field of external human–machine interfaces for autonomous vehicle-to-pedestrian communication. In the great majority of covered studies, participants clearly benefited from the presence of a communication interface when interacting with an autonomous vehicle. Nevertheless, standardized interface evaluation procedures and optimal interface specifications are still lacking.

## Introduction

Road use is officially regulated by traffic laws and standardized signals, both vehicle-based (e.g., turn signals, hazard lights, horns) and infrastructure-based (e.g., traffic lights, traffic signs, road surface markings). However, it is often the case that informal communicative cues are employed by traffic participants to further enhance traffic flow and ensure safety on the road for all parties involved (Färber, 2016; Rasouli et al., 2018). Negotiating traffic, signaling intention, resolving ambiguities, acknowledging the presence of other road users, rebuking transgressions, and even surviving reckless road behavior, are often made possible through use of hand gestures, facial expressions, and eye contact, by motorists, cyclists, and pedestrians alike (Guéguen et al., 2015, 2016; Ren et al., 2016; Dey and Terken, 2017; Sucha et al., 2017; Nathanael et al., 2018; Rasouli and Tsotsos, 2018).

In the near future, however, when semi- and fully autonomous vehicles are introduced into the traffic system, drivers will gradually assume the role of mere passengers, who are casually engaged in non-driving-related activities and, therefore, unavailable to participate in traffic interaction. This technological transformation of the traffic environment will most probably be accompanied by a social one, as no informal communication channel between drivers and pedestrians will be there to serve as an alternative to official rules and regulations or standardized signals (Stanciu et al., 2018). In this mixed-autonomy traffic environment, where manually driven, semi-, and fully autonomous vehicles operate simultaneously, advanced communication interfaces will need to be developed that inform pedestrians of the current state and future behavior of an autonomous vehicle, in order to maximize safety and efficiency for all road users, as well as enhance trust in and acceptance of the new technology (Coeugnet et al., 2018; Habibovic et al., 2018).

Human–machine interfaces that utilize the external surface and the immediate surroundings of the vehicle have been proposed as a possible solution to the communication problem road users will soon face in their attempts to interact with autonomous vehicles (Haeuslschmid et al., 2016; Colley et al., 2017; Mirnig et al., 2017). Ideally, an interface of this type would communicate information concerning vehicle driving mode (e.g., manual, semi- or fully autonomous), imminent vehicle maneuvers (e.g., yielding, taking off or changing lanes), perception of vehicle surroundings (e.g., detection of nearby pedestrians), and cooperation capabilities (e.g., ability to communicate mode or intention) (Owensby et al., 2018; Schieben et al., 2018). Furthermore, providing explicit advice or instruction to act to pedestrians would be avoided, as circumstances may warrant different actions appropriate for different pedestrians interacting with the vehicle at the same time (Habibovic et al., 2018). The relevant information would be intelligible, unambiguous, and perceptible under various environmental conditions without being distracting, while the interface would be scalable, in order to support communication with multiple road users simultaneously (Holländer, 2018, 2019; ISO/TR 23049:2018, 2018; Mirnig et al., 2018). Importantly, in the case of interfaces where communication is materialized via the windshield, it has been suggested that the most critical information be presented on its right side – the driver side from an external viewer’s perspective in a right-hand traffic environment – as it is the more readily attended side by pedestrians, especially at shorter vehicle distances (Liu et al., 2017; Dey et al., 2019). Finally, according to Werner (2019), turquoise would be the most appropriate color to utilize in light-based autonomous vehicle-to-pedestrian communication, due to its saliency, discriminability, attractiveness, and uniqueness in the traffic system.

During live demonstrations of a Level 4 autonomous vehicle (SAE International, 2016), Merat et al. (2018) measured pedestrians’ and cyclists’ attitudes toward the new technology. Participants were first given the opportunity to interact with the vehicle in shared or dedicated space, i.e., in the absence or presence of a designated lane for its movement, and then provided relevant feedback as well as suggestions on possible interface implementations. Results showed that the presence of a designated lane enhanced the feeling of safety when interacting with the vehicle. Notably, with regard to the ideal implementation, receiving information about whether they had been detected was prioritized over information concerning vehicle intentions, while lights and sounds were preferred to written and spoken language for communicating information about vehicle intentions and detection of vulnerable road users, i.e., motorcyclists, cyclists, pedestrians, elderly, disabled people, and children. Interestingly, in a focus group with children aged 7–10 years, aimed at addressing child-pedestrian needs when interacting with autonomous vehicles, Charisi et al. (2017) identified the need to accurately recognize that a vehicle is in autonomous mode, as well as the need to interact with design metaphors that are based on children’s existing mental models and own experiences in traffic.

Numerous physical prototypes have been developed by automotive manufacturers, technology companies, and research groups operating in academia. For instance, Drive.ai^1^ utilizes LED (light-emitting diode) panels, positioned on the hood, over the front fenders, and on the rear of the vehicle, to communicate vehicle mode and intention to other road users. When in manual mode, all panels display “Person Driving” accompanied by a chauffeur illustration. When yielding to a pedestrian, “Waiting for you to cross” is displayed on the side panels, accompanied by the pedestrian-crosswalk sign, while “Pedestrian crossing” is displayed on the rear panel. Jaguar Land Rover^2^ utilizes anthropomorphic design to communicate vehicle intentions to pedestrians. The headlights, serving as the “eyes” of the vehicle, seek to make eye contact with nearby pedestrians to acknowledge their presence and signal the vehicle’s intention to yield to them. Lyft’s^3^ notification system turns vehicle windows into screens for communicating intention and advice to pedestrians and drivers via text (“yielding”; “warning: turning left”; “warning: turning right,” “safe to cross”; “safe to pass”), while the name of a future passenger can be displayed also, to enable efficient pick-up. Mitsubishi^4^ has developed an indicator system that communicates vehicle intentions, namely intended path, emergency stops, and door openings, via color light animations projected onto the road surface. Renault^5^ utilizes a frontal LED light strip to communicate vehicle mode and presence to pedestrians and cyclists. Semcon^6^ has developed the “Smiling Car,” an interface that communicates vehicle intention via a universally recognized facial expression: the smile. When the vehicle detects a pedestrian, and intends to yield to them, a smile lights up on a frontal LED display to signal that it is safe to cross. Volvo^7^ combines targeted ultrasonics with color light animations, to capture the attention of vulnerable road users and effectively communicate vehicle intentions.

As for academic research in the field, Florentine et al. (2016) have developed a notification system aimed at alerting pedestrians to the presence of an autonomous vehicle and communicating acknowledgment from the vehicle. Their interface utilizes a speaker system to capture attention via music, and a LED light strip to signal detection via changes in light color. Similarly, Benderius et al. (2018) have developed an interface that communicates an autonomous vehicle’s intended movement trajectory, distance from a desired position, and proximity to other road users, by means of a LED light strip and a speaker system.

However, all aforementioned prototypes have either not been evaluated via controlled studies employing human participants, or if they have, their findings have not been made publicly available. The aim of the present review is to provide a comprehensive account of published empirical work in the field of external human–machine interfaces for autonomous vehicle-to-pedestrian communication (for a review on vehicle-to-pedestrian communication interfaces employing mobile or wearable devices and infrastructure-based communication technology (e.g., cellular or wireless), see Sewalkar and Seitz, 2019). The included empirical studies were mainly collected through a manual search of the Google Scholar database, using the following search terms: “external human–machine interface(s),” “external HMI(s),” “vehicle-to-pedestrian communication,” and “vehicle-pedestrian communication.” We also conducted a snowball search and a citation search to identify additional studies of interest (Table 1). The sole criterion that had to be met for a study to be included in the review, was the evaluation of the interface(s) to have occurred in the context of a controlled study employing human participants. Therefore, implementations that were either only evaluated by their developers or not at all, were excluded, as was conceptual work in the field.

**TABLE 1:** Empirical studies in the field of external human–machine interfaces for autonomous vehicle-to-pedestrian communication.

Studies	Stimulus delivery			Interface parameters						Evaluation procedures			Measures	
	Physical Prototype	Monitor-based	VR- based	Technology	Location	Content type	Information type	Message coding	Modality	Behavioral task	Online survey	Questionnaire	Objective	Subjective
Hensch et al. (2019)	✓			Display	Roof	Information	Mode, intention	Lights	Visual	Intention identification		Comprehensibility, trust, safety, usefulness		Likert scales, interview
Costa (2017)	✓			Cardboard, speaker	Hood, bumper	Advice		Textual, pictorial, sounds	Visual, auditory	Street-crossing			Frequency	
Mahadevan et al. (2018)	✓			Light strip, display, LEDs, printed hand, mobile phone, speaker	Windshield, hood, roof, street surface, pedestrian’s mobile phone	information	Pedestrian acknowledgment, intention	Lights, speech, vibration, gesture, pictorial	Visual, auditory, haptic	Crossing intention		Effectiveness, confidence		Likert scales, interview
Habibovic (2018)	✓			Light strip	Windshield	Information	Mode, intention	Lights	Visual	Street-crossing		Safety		Likert scales, interview
Clamann et al. (2017)	✓			Display	Radiator grille	Information, advice	Speed	Textual, pictorial	Visual	Street-crossing		Effectiveness	Decision time	Interview
Li et al. (2018)		✓		Display	Windshield, radiator grille, vehicle sides	Advice		Lights	Visual		Situational urgency, crossing intention			Numeric scales, interview
Zhang et al. (2017)		✓		Light strip	Front doors, hood	Information	Intention	Lights	Visual		Intention identification, effectiveness			Interview
Song et al. (2018)		✓		Display	Radiator grille	Advice		Textual, pictorial	Visual		Crossing intention, preference		Reaction time, frequency	interview
Fridman et al. (2017)		✓		Light strip, display, projection, vehicle lights and signals	Windshield, headlights, fog lights, directional signals, radiator grille, bumper, street surface	Information advice	Intention	Textual, pictorial, lights	Visual		Crossing intention		Error rates, reaction time	
Ackermann et al. (2019)		✓		Light strip, display, projection	Windshield, radiator grille, street surface	Information, advice	Mode	Lights, textual, pictorial	Visual			Comprehensibility, recognizability, ambiguousness, comfort		Numeric scales, interview
Petzoldt et al. (2018)		✓		Light strip	Above license plate	Information	Deceleration	Lights	Visual	Deceleration detection		Usefulness, safety	Error rates, reaction time	Likert scales
Chang et al. (2018)		✓		Light strip, display, projection, rotating vehicle lights	Windshield, radiator grille, street surface, headlights	Information	Intention	Lights, textual, pictorial, anthropomorphism	Visual	Intention identification		Intelligibility	Error rates	Likert scales
Charisi et al. (2017)		✓		Display, light strip, projection, vehicle lights and signals	Windshield, headlights, directional signals, street surface	Information	Intention	Lights, textual, pictorial, anthropomorphism	Visual	Intention identification		Intention identification	Error rates	Interview
de Clercq et al. (2019)			✓	Display, vehicle lights and signals	Radiator grille, frontal brake lights	Information advice	Intention	Textual, lights, pictorial	Visual	Safety-reporting		Safety, preference	Duration	Interview
Hudson et al. (2018)			✓	Display, speaker	Hood	Advice		Textual, pictorial, speech, music	Visual, auditory	Street-crossing		Preference		Interview
Deb et al. (2018)			✓	Display, speaker	Hood	Information advice	Intention	Lights, pictorial, speech, sounds, music	visual, auditory	Street-crossing		Safety, acceptance	Decision time, duration	Likert scales, interview
Stadler et al. (2019)			✓	Display	Radiator grille	Advice		Lights, textual, pictorial,	Visual	Street-crossing		Satisfaction	Error rates, decision time	Numeric scales, interview
Othersen et al. (2018)			✓	Display	Radiator grille	Information	Pedestrian detection, intention	Lights, pictorial	Visual	Street-crossing		Effectiveness, understandability, perceptibility, safety, appeal	Decision time	Interview
Chang et al. (2017)			✓	Rotating vehicle lights	Headlights	Information	Pedestrian acknowledgment, intention	Anthropomorphism	Visual	Crossing intention		Effectiveness, safety	Error rates, reaction time	Likert scales, interview
Böckle et al. (2017)			✓	Light strip, speaker	Vehicle corners	Information	Intention	Lights, sounds	Visual, auditory	Street-crossing		Safety, comfort, effectiveness	Decision time	Likert scales, interview

## External Human–Machine Interfaces Evaluated Via Empirical Studies

### Differences in Methodology

Ideally, in the context of an empirical study, a fully functional physical prototype of an interface would be evaluated under real-world traffic conditions, in order to maximize the possibility for environmental generalization of research findings. However, this has never been the case so far, mainly due to considerations regarding feasibility and safety. Physical prototypes require vast amounts of resources to develop, while autonomous vehicles have not been allowed into regular traffic yet. The few studies that have actually evaluated physical prototypes of interfaces under real-world traffic conditions, have managed to do so by employing the Wizard of Oz technique, where autonomy is merely simulated, while a human operator is concealed inside the vehicle, and only after the evaluation procedure are participants informed of the relevant manipulation (Habibovic et al., 2016). The main advantage of this technique is that it allows for *in situ* observation and measurement of pedestrian behavior when interacting with a supposedly autonomous vehicle, outside the confines of a typical psychology laboratory (Rothenbücher et al., 2016). Nevertheless, the majority of studies have been conducted in laboratory settings, utilizing either desktop computers or virtual reality (VR) pedestrian simulators for the presentation of experimental stimuli and the collection of behavioral data. While traffic scenarios experienced in monitor-based studies may lack realism to a great extent, they do allow for rapid prototyping, and they provide greater safety to participants. In addition, monitor-based studies provide greater flexibility in parameter manipulation and greater experimental control to researchers, compared to studies employing physical prototypes. VR-based studies, on the other hand, manage to effectively combine the advantages of typical monitor-based studies with an added sense of realism, due to the immersive nature of the technology (Deb et al., 2017).

### Studies Employing Physical Prototypes

At one extreme of the ecological validity continuum, one will find studies that have employed physical prototypes of communication interfaces in evaluation procedures occurring under real-world traffic conditions. A case in point are Hensch et al. (2019), who evaluated an interface, developed to communicate mode and intention of an autonomous vehicle, with regard to its effectiveness in imparting feelings of safety and comfort to pedestrians interacting with the vehicle. Their interface consisted of a LED display, positioned on the vehicle roof, conveying three different messages via color and light-motion combinations. “Autonomous mode” was communicated via a constantly lit, turquoise light bar, whereas “vehicle approaching” was communicated via the light bar flashing, and “yielding” via a continuous, sweeping movement of the light bar across the LED display. In a parking area, random pedestrians interacted with a vehicle – autonomous or manually driven – equipped with the interface, and were interviewed immediately thereafter. The majority of participants reported feeling safer interacting with the manually driven vehicle compared to the autonomous vehicle, regardless of presence or absence (baseline condition) of the interface. Moreover, the interface was found to be intuitively incomprehensible and only partially trustworthy. However, the general usefulness of external interfaces for communicating mode and intention was noted by the majority of participants.

In like manner, Costa (2017) evaluated an interface developed to support effective street-crossing when interacting with an autonomous vehicle. The interface consisted of two plastic cardboards, one displaying text (“Please go” in green; “Stop” in red), and one displaying an icon (pedestrian silhouette in green; raised hand in red), positioned on the right side of the hood and the right side of the bumper, respectively, as well as a speaker system emitting standard traffic-light sounds (fast tempo for crossing; slow tempo for not crossing), positioned behind the cardboards. Random pedestrians interacted with an autonomous vehicle equipped with the interface at an unsignalized crosswalk. Results showed that, given the absence of visual feedback from the driver, pedestrians were more confident to cross the street when the vehicle explicitly communicated its intention to give right of way, compared to the baseline condition (autonomous vehicle without interface). Accordingly, when the vehicle explicitly communicated its intention to not give right of way, pedestrians were more hesitant to cross the street, compared to the baseline condition.

However, results from Hensch et al. (2019) and Costa (2017) should be interpreted with caution, as in both studies random pedestrians served as participants, leaving room for doubt with respect to the possibility for population generalization of findings. On the contrary, in all other field studies covered here, participants have been screened according to various criteria (e.g., pedestrian experience, visual acuity, mobility impairment, age), and their performance in a well-defined behavioral task (e.g., specifying intention to or actually crossing a street) has been carefully measured. For example, Mahadevan et al. (2018) evaluated four interfaces aimed at acknowledging pedestrian presence and signaling vehicle intention, by measuring participants’ crossing intention. Designs utilized one or more modalities (visual; auditory; haptic) and locations (windshield; hood; roof; street surface; pedestrian’s mobile phone), to present relevant information. More specifically, the “vehicle-only” interface consisted of a LED light strip and a speaker, positioned on the windshield and the hood, respectively. Awareness of a pedestrian was communicated via blinking blue lights, whereas, intention was communicated via solid red (“not stopping”), green (“stopping”), and yellow (“starting”) lights, and accompanying verbal messages (“stopping”; “starting”). The “vehicle and street infrastructure” interface consisted of a speaker and three LEDs, positioned on the hood and onto the street surface, respectively. Awareness was communicated via a verbal message (“I see you”), whereas intention was communicated via solid red (“not stopping”), green (“stopping”), and yellow (“starting”) lights, and accompanying verbal messages. The “vehicle and pedestrian” interface consisted of a display, positioned on the front of the vehicle, and an Android phone held by the pedestrian. Awareness was communicated via an animated face, looking straight ahead initially, and then directing its gaze to the pedestrian, whereas, intention was communicated via phone vibration. Finally, the “mixed” interface consisted of three LEDs and a printed hand, positioned onto the street surface and on the vehicle roof, respectively, as well as an Android phone held by the pedestrian. Awareness was communicated via a verbal message (“I can see you”) emitted by the phone, whereas, intention was communicated via the three LEDs and the actuated hand producing a waving gesture (“stopping”). In a parking garage, participants were tasked with reporting their intention to cross the street, while an autonomous vehicle equipped with one of the interfaces was approaching. Results showed that receiving explicit information via an interface was preferred to receiving only implicit information via vehicle kinematics (i.e., distance and speed) in the baseline condition (autonomous vehicle without interface). Also, participants rated information about vehicle intention as more important than information about their acknowledgment. In terms of effectiveness, the “vehicle and street infrastructure” interface was rated as the most effective, whereas, the “vehicle and pedestrian” interface was rated as the least effective.

Similarly, Habibovic (2018) evaluated the “Autonomous Vehicle Interaction Principle” (AVIP), an interface developed to convey information about an autonomous vehicle’s mode and intention, by measuring pedestrians’ perceived safety. Their interface consisted of an RGB (red, green, blue) LED light strip positioned at the top of the windshield, that conveyed three different messages via color (white/yellow) and light-motion combinations. “I am in automated mode” was signaled by the constantly lit middle part of the strip, “I am about to yield” was signaled by the lit middle part gradually expanding to cover the whole strip, and “I am about to start driving” was signaled by the exact opposite movement, until only the middle part was lit again. In a parking garage, participants were tasked with crossing a street in front of a vehicle – autonomous or manually driven – that was either approaching or standing still. In both moving- and stationary-vehicle conditions, participants reported feeling safer when interacting with the manually driven vehicle compared to the autonomous vehicle. However, perceived safety was greater when interacting with an autonomous vehicle equipped with the AVIP compared to the baseline condition (autonomous vehicle without interface). Interestingly, in the presence of the AVIP, participants reported feeling as safe as when interacting with the manually driven vehicle.

While the aforementioned studies have used mainly subjective measures to assess interface effectiveness, Clamann et al. (2017) evaluated a communication interface by using an objective measure, namely decision time, alongside ratings and interviews. Their interface consisted of a LED display positioned on the radiator grille of a vehicle, displaying in black and white either informative content (current speed) or advisory content (a pedestrian crossing sign, communicating crossing is allowed; a crossed out pedestrian crossing sign, communicating crossing is not allowed). Participants were asked to either cross a street at an unsignalized crosswalk or jaywalk (i.e., cross the street unlawfully, at a place where it is prohibited), while an autonomous vehicle equipped with the interface was approaching (24 km/h; 40 km/h). No effect of interface on street-crossing efficiency was found, as evidenced by decision times (i.e., time between looking at the display and initiating crossing). In support of this finding, only a small minority of participants reported being influenced by the interface in their decision-making. Most participants reported distance from and speed of the vehicle to have determined their crossing behavior.

Evidently, results from studies employing the Wizard of Oz technique to evaluate physical prototypes of communication interfaces do not paint a clear picture regarding the potential of the proposed solution to the impending traffic interaction problem. On the one hand, participants preferred interacting with manually driven vehicles to interacting with autonomous vehicles, and based their decision-making on vehicle kinematics. This comes as no surprise and can be greatly attributed to unfamiliarity with both the autonomous technology and the concept of a communication interface, as well as to safety concerns, accentuated by the fact that all studies were conducted under real-world traffic conditions. On the other hand, in interactions with autonomous vehicles, external human–machine interfaces were acknowledged as an acceptable substitute to explicit driver feedback, and a desirable additional source of information, complementary to vehicle kinematics, lending hope to the possibility that they may indeed be an appropriate solution to the problem at hand.

### Monitor-Based Studies

At the other extreme of the ecological validity continuum, sit studies that have utilized desktop computers in evaluation procedures occurring under artificial conditions. Generally speaking, monitor-based studies in the field of autonomous vehicle-to-pedestrian communication have come in two varieties: online surveys and laboratory experiments. In the case of online surveys, a crowdsourcing approach is usually adopted for participant recruitment, and participants are typically asked to perform a behavioral task and/or complete a questionnaire from a location of their choice. In one such study, Li et al. (2018) evaluated an interface with respect to its potential to communicate situational urgency to pedestrians in the event of a fast-approaching autonomous vehicle. Their interface consisted of light displays positioned at the top of the windshield, close to the bottom of each side of the vehicle, and on top of the radiator grille, that conveyed different messages contingent on the distance between moving vehicle and pedestrian. Two different designs were evaluated: in the first, “safe to cross,” “safe, but not recommended,” and “dangerous to cross” messages were signaled by a solid green light, a flashing yellow light, and a solid red light, respectively, whereas, in the second, they were signaled by a solid white light, a flashing red light, and a solid red light, respectively. Participants watched animated videos of the approaching vehicle (50 km/h, constant speed; vehicle decelerating), and were asked to rate perceived urgency for each design, while considering the pedestrian perspective, as well as to indicate likelihood of crossing the street. Results showed that both designs were perceived as more urgent compared to the baseline condition (autonomous vehicle without interface), as were flashing-color warnings compared to solid-color warnings. However, the majority of crossing decisions was found to be based on vehicle kinematics and not on the interface.

In the context of another online survey, Zhang et al. (2017) evaluated the “Intention Indicator,” an interface developed to facilitate traffic flow at four-way stop intersections, by indicating the intentions of an autonomous vehicle to other road users. Their interface consisted of an RGB LED light strip positioned on the front doors and hood of the vehicle, and was designed to communicate five vehicle intentions via different color and light-motion combinations. Vehicle intentions, namely “slowing down,” “waiting,” “planning to go,” “starting to go,” and “going,” were signaled by a forward-moving green light, a static green light, a flashing white light, a slowly backward-moving red light, and a fast backward-moving red light, respectively. Participants watched videos of the vehicle equipped with the interface, and were asked to identify the communicated intention, as well as rate the effectiveness of various alternative color and light-motion combinations in communicating vehicle intentions. Results showed that participants clearly perceived the interface as communicating vehicle intention, and not instructions or advice to other road users. However, “planning to go” was confused with “waiting,” and “starting to go” was confused with “going.” Participants preferred green color for indicating “starting to go” and “going,” and red color for indicating “slowing down” and “waiting.” Finally, forward-moving lights were preferred for indicating a moving or accelerating vehicle, whereas backward-moving lights were preferred for indicating a stopping or decelerating vehicle.

Whereas Li et al. (2018) and Zhang et al. (2017) studied interface effects on perceived situational urgency and vehicle intention identification, respectively, Song et al. (2018) and Fridman et al. (2017) focused on street-crossing performance instead. More specifically, Song et al. (2018) developed an interface to study the effect of content type on jaywalking decisions. Their interface was positioned on the radiator grille of the vehicle, and consisted of two monitors: a right monitor displaying the zebra crossing sign, and a left monitor displaying text that reassured pedestrians it was safe to cross, either in the form of an affirmative statement (“OK!”) or in the form of a command (“GO!”). Participants viewed real-world videos of an approaching autonomous vehicle equipped with the interface, and made speeded jaywalking decisions. Results revealed no effect of content type on crossing frequency, efficiency, or subjective evaluation of the interface. Compared, however, to the baseline condition (autonomous vehicle without interface), participants crossed the street more often when the approaching vehicle was equipped with the interface.

In like manner, Fridman et al. (2017) evaluated 30 interfaces with respect to their potential for communicating vehicle intentions to pedestrians. Interfaces utilized several locations (windshield; headlights; fog lights; directional signals; radiator grille; bumper; street surface), while intention was communicated via text (“WALK”; “DON’T WALK”; “GO”; “STOP”; “CAR STOPS”), icons (walking silhouette; raised hand; STOP traffic sign; pedestrian sign; directional arrows; dotted circle), and light color (green; red; yellow; white). Participants viewed augmented real-world photos of a vehicle equipped with one of the interfaces approaching an unsignalized intersection, and were asked to make crossing decisions. As far as designs communicating crossing was safe are concerned, the walking silhouette displayed on the windshield in green, “WALK” displayed on the windshield in green, the directional arrows projected onto the street surface in front of the vehicle in green, and “WALK’ projected onto the street surface in front of the vehicle in green, were the most accurately responded to. With regard to designs suggesting crossing was not safe, the raised hand displayed on the windshield in yellow, and “DON’T WALK” displayed on the windshield in red, were the most accurately responded to.

It is worth mentioning that the popularity of online surveys in psychological research has been steadily increasing, as they have proven to be extremely efficient with relation to participation and remuneration. However, due to the very nature of the procedure, attentional engagement with the task in hand on the part of the participant cannot be ensured to the extent it can in the context of a laboratory experiment. Accordingly, results from the aforementioned online surveys should also be interpreted with caution.

On the subject of monitor-based studies conducted in laboratory settings, the approach has been adopted for interface evaluation in four cases. In one of them, Ackermann et al. (2019) studied the effect of four interface parameters on intuitive comprehensibility, recognizability, ambiguousness, and interaction comfort, as experienced by pedestrians. The interfaces differed in technology used (projection; LED display; LED light strip), location (windshield; radiator grille; street surface), message coding (pictorial: car icon, directional arrows; textual: “Automatic mode,” “Go ahead”), and content type (vehicle mode information; advice to pedestrian). Participants viewed augmented real-world videos of an autonomous vehicle approaching equipped with one of the interfaces (20 in total), and were asked to rate them, while considering the pedestrian perspective. In terms of intuitive comprehensibility, LED light strips received the lowest rating. As far as recognizability is concerned, projections were better recognized than LED displays, as was advice to pedestrian compared to vehicle mode information, irrespective of message coding. Moreover, vehicle mode information was rated as more ambiguous compared to advice to pedestrians, as was pictorial coding compared to textual coding. Finally, projections and advice to pedestrian were rated as more comfortable to interact with, compared to LED displays and vehicle mode information, respectively.

In typical experimental fashion, Petzoldt et al. (2018) evaluated an interface developed to communicate information about vehicle deceleration. Their interface consisted of a frontal brake light positioned above the front license plate, that lit up green as soon as the vehicle started to decelerate. Participants viewed real-world videos of the vehicle approaching, and made speeded judgments about vehicle deceleration. Results showed that the interface facilitated deceleration detection, as evidenced by shorter reaction times compared to the baseline condition (vehicle without interface). Interestingly, once participants had become familiar with the interface and had experienced its usefulness, they were slower to detect deceleration without its assistance. Additionally, the majority of participants noted the potential of the interface to increase pedestrian safety and prevent crashes.

Furthermore, Chang et al. (2018) compared five existing interfaces, developed by automotive manufacturers, technology companies, and research groups, to communicate the intentions of an autonomous vehicle to other road users. In the first interface, the headlights, serving as the vehicle’s “eyes”, turned to look at a pedestrian on the sidewalk, and offered right of way by slowly moving horizontally across the other side of the street. In the second interface, on a LED display positioned on the radiator grille, an orange straight line turned into a smile to signal that the vehicle was yielding to the pedestrian. In the third interface, on a LED display positioned on the radiator grille, text in orange (“You Can Cross”) prompted the pedestrian to cross. In the fourth interface, a LED light strip positioned on the bottom of the windshield, emitted a flashing green light to signal that it was safe to cross. Finally, in the fifth interface, the image of a crosswalk was projected onto the street surface in front of the vehicle, to assist the pedestrian in crossing the street. Participants watched animated videos of an autonomous vehicle equipped with one of the interfaces approaching an unsignalized crosswalk, and were tasked with making judgments about vehicle intentions regarding yielding. The textual interface was the most accurately responded to, followed by the projected-crosswalk interface. It also ranked first in intelligibility, followed again by the projected-crosswalk interface.

Although, similar to Chang et al. (2018), interface effects on vehicle intention identification were the focus in Charisi et al. (2017), children aged 7–10 years served as participants in their case. More specifically, a number of interfaces were evaluated with regard to their potential for effectively addressing child-pedestrian needs when interacting with autonomous vehicles. Interfaces utilized traffic lights, traffic signs, projected crosswalks, drawings of children holding “GO” or “STOP” placards, pedestrian figures, headlights, LED light strips, and vehicle anthropomorphism, to communicate vehicle intention with respect to yielding to a child pedestrian. A picture questionnaire was administered to child participants, who were tasked with reporting right of way according to each design depiction. Results showed that already familiar designs, namely traffic lights and signs, as well as novel designs based on existing mental models, like drawings of children holding “GO” or “STOP” placards, were the most accurately recognized. The anthropomorphized vehicle, on the other hand, was the least recognizable design. Moreover, standard traffic colors (red, green, orange) were more accurately recognized than colors not commonly used in traffic regulation (purple, dark blue, light blue).

Compared to studies employing physical prototypes, results from monitor-based studies that actually tested for the effect of equipping a vehicle with an external human–machine interface, provide stronger evidence of the usefulness of the concept. Interfaces appear to have facilitated situational urgency communication, street-crossing decision-making, as well as vehicle deceleration detection. Although these findings may partly be attributed to the reassurance the safe confines of a typical psychology laboratory or their own personal environment can provide to participants, the utilization in most cases of an objective measure, namely reaction time or accuracy, points toward a genuine positive effect of interface.

### VR-Based Studies

Situated around the middle of the ecological validity continuum, one will find studies that have utilized VR pedestrian simulators in the interface evaluation procedure. As already mentioned, these studies manage to combine the best of both worlds, i.e., tight experimental control, nevertheless, under highly realistic conditions. In the context of one such study, de Clercq et al. (2019) evaluated four interfaces, developed to communicate yielding intention, with respect to their effect on perceived safety when interacting with an autonomous vehicle. The interfaces – all employing the radiator grille area of the vehicle – were: a set of frontal brake lights (light cyan when yielding; green when not yielding), the “Knightrider” (a short animated bar, repeatedly moving from left to right when yielding, otherwise, remaining fixed in the center), the “Smiley” (a long horizontal line, curving to resemble a smile when yielding, and remaining straight when not yielding), and text in light cyan (“WALK”; “DON’T WALK”). Participants were tasked with indicating their intention to jaywalk in front of an approaching autonomous vehicle equipped with one of the interfaces, while at the same time providing information concerning the temporal unfolding of their feeling of safety, by holding a button pressed for as long as they felt safe to actually cross. Results showed that, in the yielding condition, the temporal window of perceived safety was wider (i.e., participants felt safe to cross the street for a longer period of time) when they encountered an autonomous vehicle equipped with an interface compared to the baseline condition (autonomous vehicle without interface). However, the presence of an interface had no effect on the duration of the feeling of safety when the autonomous vehicle intended not to yield.

In another VR-based study, Hudson et al. (2018) studied the effect of passenger status (attentive driver; inattentive driver; no driver) on the ratings of an interface developed to support pedestrians in their interactions with autonomous vehicles. Their interface consisted of a LED display, positioned on the hood of the vehicle, displaying either text in green (“WALK”), a white walking silhouette, a red raised hand or a STOP sign, and of a speaker system, playing either music or a verbal message (“safe to cross”). Participants were tasked with crossing the street at an unsignalized crosswalk, while an autonomous vehicle equipped with the interface was approaching. Results showed that the interface was preferred to the baseline condition (autonomous vehicle without interface). The text and the STOP sign were the highest rated visual designs, whereas the verbal message was the preferred audio design. As for the effect of passenger status on preference, interfaces that were installed on a vehicle featuring either an attentive driver or no driver at all were preferred to interfaces installed on a vehicle featuring an inattentive driver.

While de Clercq et al. (2019) and Hudson et al. (2018) studied interface effects on perceived safety duration and preference, respectively, the following studies have rather focused on street-crossing performance. For example, Deb et al. (2018) evaluated a number of interfaces consisting of a visual and/or an audible feature, with regard to their ability to impart a feeling of safety to pedestrians interacting with autonomous vehicles, influence their crossing behavior, and increase their acceptance of the new technology. Visual features, displayed on the hood of the vehicle, included flashing text (“BRAKING”) in green, an animated white pedestrian silhouette, and a flashing smile in green, whereas, audible features included a horn sound, music, and a verbal message (“safe to cross”). Participants were tasked with crossing in front of an autonomous vehicle that was yielding to them at an unsignalized crosswalk. In terms of perceived safety, all interfaces were preferred to the baseline condition (autonomous vehicle without interface). Additionally, the flashing text and the animated silhouette were the highest rated visual features, whereas, the verbal message was the preferred audible feature. Interestingly, the older age groups (31–40; 40+) found interfaces to be more useful than younger participants (18–30) did. As far as waiting and crossing times are concerned, music and the verbal message led to the shortest crossing times, whereas, the horn sound led to the longest crossing times, even longer than the baseline condition. Finally, equipping an autonomous vehicle with a communication interface was found to have a positive effect on acceptance of the new technology.

Similarly, Stadler et al. (2019) evaluated an interface with respect to its effectiveness, efficiency, and user satisfaction, when utilized to assist pedestrians in crossing the street in front of an autonomous vehicle. The radiator grille area of the vehicle was employed for the display of designs, that included human silhouettes (walking green; standing red), traffic lights (green; red), LED light strips (green; red), icons (directional green arrows; raised red hand), and marks (check mark; “X” mark). Participants were tasked with jaywalking in front of an approaching autonomous vehicle equipped with the interface. All designs proved to be efficient, as evidenced by shorter decision times when compared to the baseline condition (autonomous vehicle without interface). Additionally, all designs were found to be effective, as evidenced by lower error rates when compared to the baseline condition. LED light strips, however, were found to be the least effective. With regard to user satisfaction, all designs were rated as more satisfactory than the baseline condition. The highest overall satisfaction was reported for the icons, as they were the most detectable, comprehensible, influential, and cognitively undemanding design, whereas the lowest user satisfaction was reported for the LED light strips.

The main focus in Othersen et al. (2018) was the effect of two interface parameters, namely message coding (abstract; pictorial) and dynamics (static; animated), on communication effectiveness and street-crossing efficiency. Their interface utilized the radiator grille area of an autonomous vehicle, and employed four different designs. Pedestrian detection was communicated via either a light bar, that lit up at a distance of 50 m from the pedestrian and deactivated after they had crossed, or a drawing of an eye. Yielding intention was communicated via either a light bar, performing a continuous, sweeping movement across the radiator grille area, or an animation of a walking pedestrian accompanied by directional arrows. Participants were asked to make street-crossing decisions, while an autonomous vehicle equipped with the interface was approaching. All designs proved to be efficient in supporting street-crossing decisions, as evidenced by shorter crossing initiation times to vehicle stop, i.e., time between vehicle coming to a full halt and pedestrian initiating crossing, when compared to the baseline condition (autonomous vehicle without interface). This effect was especially pronounced in the case of the animated designs. On the contrary, only the animated designs were effective in communicating pedestrian detection and vehicle intention, according to subjective data. While the walking-pedestrian animation was rated highest in understandability, perceptibility, and appeal, the static designs were found to be uninformative, imperceptible, and unrelated to both vehicle mode and future behavior. However, in general, the possibility of equipping autonomous vehicles with external interfaces for communicating pedestrian detection and vehicle intention was positively evaluated.

Moreover, Chang et al. (2017) evaluated the “Eyes on a Car,” an interface where the headlamps served as the “eyes” of the autonomous vehicle, in order to substitute for the lack of eye contact between driver and pedestrians. When the vehicle intended to yield, the headlamps turned and looked at the pedestrian to acknowledge their presence and communicate the vehicle’s intention; otherwise, the headlamps kept looking straight ahead, along the road. Participants were asked to make speeded crossing decisions at an unsignalized crosswalk, while an autonomous vehicle was approaching. The majority of participants reported feeling safer crossing in front of a vehicle that was equipped with “Eyes on a Car” compared to the baseline condition (autonomous vehicle without interface), and that the interface assisted them in their decision-making. In support of these findings, reaction times were shorter in the presence of the interface compared to the baseline condition.

Finally, Böckle et al. (2017) evaluated the “SAV2P” (Shared Automated Vehicle to Pedestrian), an interface developed to communicate the intentions of a shared automated vehicle, with regard to its potential for enhancing perceived safety and comfort of pedestrians interacting with the vehicle. Their interface employed LED columns, positioned on each of the four corners of the vehicle, and a speaker system for conveying relevant messages via color, light motion, and sound. “Not stopping” was signaled by a flashing yellow light, “stopping” by a vertically moving blue light, “waiting” by a slowly fading blue light, and “start driving” by a flashing yellow light accompanied by a bell sound. Participants were tasked with crossing the street at an unsignalized crosswalk, while a shared automated vehicle was yielding to them. Overall, they reported feeling safer and more comfortable crossing the street in front of the vehicle when the interface was switched on compared to when it was switched off, and that the interface assisted them in their crossing decisions. In agreement with these findings, behavioral data revealed that participants were more hesitant to initiate crossing in the absence of the interface.

Admittedly, in the case of VR-based studies, there is unanimous evidence of the usefulness of external human–machine interfaces in autonomous vehicle-to-pedestrian communication. Across all studies, interfaces were found to have facilitated street-crossing decision-making, and/or to have led to higher perceived safety, higher acceptance of the autonomous technology, and a more positive traffic interaction experience. Considering these results were obtained under highly realistic conditions via mainly objective measures, without compromising experimental control, they can be taken to clearly demonstrate a genuine positive effect of interface.

## Discussion

In the mixed-autonomy traffic environment of the near future, traffic participants will not be able to rely on informal communication channels to facilitate their interactions to the extent they currently do. The promise of autonomous vehicles for less traffic congestion, less traffic accidents, and, most importantly, less traffic fatalities, will most probably be delivered accompanied by limitations on the available communication channels for interacting with them. However, for this new technology to be initially trusted and eventually accepted by all road users, it will need to be safe, efficient, and easy to interact with.

The empirical studies covered here evaluated external human–machine interfaces specifically developed to substitute for the lack of driver feedback, with the aim of supporting pedestrians in safely, efficiently, and easily interacting with autonomous vehicles. Across the great majority of studies, interactions with vehicles equipped with a communication interface were found to be more effective and efficient, and were perceived as safer and more satisfactory, compared to interactions with vehicles without an interface. Only in Hensch et al. (2019), Li et al. (2018) and Clamann et al. (2017) did the presence of an interface have no effect on pedestrian behavior, and crossing decisions were based on vehicle kinematics rather than explicit communicative cues.

Interestingly, the most convincing evidence were obtained largely from studies conducted in laboratory settings, namely monitor-based and VR-based studies, that utilized mainly objective measures, like reaction time, duration, and accuracy, in the context of behavioral tasks. This discrepancy between laboratory studies and studies employing physical prototypes may be attributed, at least partly, to safety concerns and measurement reliability, as the latter were conducted under real-world traffic conditions and employed mainly subjective measures, such as ratings. As has been argued elsewhere (Dey et al., 2018), best practices in the field of autonomous vehicle-to-pedestrian communication are not established yet. However, standardization of relevant procedures is a fundamental requirement for effective interface evaluations and meaningful comparisons. Therefore, future conceptual and empirical work in the field should primarily be concerned with producing standardized procedures for evaluating and comparing different implementations.

With respect to specific interface characteristics, in terms of location, the “vehicle and street” combination was rated as the most effective in Fridman et al. (2017) and Mahadevan et al. (2018), whereas street projections were rated as the most recognizable, unambiguous, and comfortable to interact with in Ackermann et al. (2019). As far as content type is concerned, advice to pedestrians was preferred to information about vehicle mode in Ackermann et al. (2019) whereas no effect of content type on pedestrian behavior was found in Clamann et al. (2017) and Song et al. (2018). With regard to information type, information about vehicle intention was rated as more important than information about pedestrian detection in Mahadevan et al. (2018) contrary to participant suggestions in Merat et al. (2018). In terms of message coding, even though lights and sounds were preferred to text and speech in Merat et al. (2018) the most accurately responded to design in Chang et al. (2018) was textual, while the preferred designs in Stadler et al. (2019) and Othersen et al. (2018) were pictorial.

It becomes easily apparent that there is yet no consensus among researchers on which specific characteristics constitute an external human–machine interface for autonomous vehicle-to-pedestrian communication effective, efficient, and usable. Some have even suggested that, in the future, autonomous vehicles could communicate with other road users via a social robot proxy positioned in the driver’s seat (Mirnig et al., 2017) however, relevant empirical work is lacking. This is to be expected considering research in the field is in its infancy, as evidenced by the fact that the earliest study included in our review was published in as recent as 2017. To make matters worse, in the majority of studies, a *ceteris paribus* approach is not chosen when it comes to measuring the effect of specific interface parameters (e.g., technology, location, content type, information type, message coding, modality) on pedestrian behavior, thus rendering direct comparisons of interfaces impossible and results inconclusive, due to confounding factors. Accordingly, outlining optimal interface specifications via proper experimental design should be the other main focus of future endeavors in the field.

To sum up, with few exceptions, participants in the covered studies clearly benefited from the presence of a communication interface when interacting with an autonomous vehicle. Nevertheless, standardized interface evaluation procedures and optimal interface specifications are still lacking.

## Author Contributions

AR contributed to conceptualization, manuscript preparation, and manuscript revision. HA contributed to manuscript revision.

## Conflict of Interest

The authors declare that the research was conducted in the absence of any commercial or financial relationships that could be construed as a potential conflict of interest.

## Footnotes

1 https://www.wired.com/story/driveai-self-driving-design-frisco-texas/

2 https://www.dezeen.com/2018/09/04/jaguar-land-rovers-prototype-driverless-car-makes-eye-contact-pedestrians-transport/

3 https://www.dezeen.com/2018/12/14/lyft-patent-notification-system-self-driving-cars/

4 https://emea.mitsubishielectric.com/en/news-events/releases/2015/1023-a/pdf/151023-2970_Road-illuminating_Directional_Indicators-G.pdf

5 https://www.dezeen.com/2018/03/06/renault-ez-go-driverless-transport-system-geneva-motor-show/

6 https://semcon.com/smilingcar/

7 https://www.slashgear.com/volvo-360c-autonomous-car-concept-flying-alternative-pedestrian-communication-05544441/
