# Corrigendum: External Human–Machine Interfaces for Autonomous Vehicle-to-Pedestrian Communication: A Review of Empirical Work

**DOI:** 10.3389/fpsyg.2020.575151

**Published:** 2020-10-16

**Authors:** Alexandros Rouchitsas, Håkan Alm

**Affiliations:** Humans and Technology Division, Luleå University of Technology, Luleå, Sweden

**Keywords:** traffic interaction, human–vehicle interaction, autonomous vehicles, vehicle-to-pedestrian communication, external human–machine interfaces, vulnerable road users

In the original article, there were five errors.

1. The word “only” was used instead of “mainly.”

A correction has been made to section External Human–Machine Interfaces Evaluated Via Empirical Studies, sub-section Studies Employing Physical Prototypes. The corrected sentence reads as follows:

“While the aforementioned studies have used mainly subjective measures to assess interface effectiveness, Clamann et al. (2017) evaluated a communication interface by using an objective measure, namely decision time, alongside ratings and interviews.”

2. The word “reaction” was used instead of “decision”.

A correction has been made to External Human–Machine Interfaces Evaluated Via Empirical Studies, sub-section VR-Based Studies. The corrected sentence reads as follows:

“All designs proved to be efficient, as evidenced by shorter decision times when compared to the baseline condition (autonomous vehicle without interface).”

3. The word “experimental” was used instead of “behavioral”.

A correction has been made to Discussion section. The corrected sentence reads as follows:

“Interestingly, the most convincing evidence were obtained largely from studies conducted in laboratory settings, namely monitor-based and VR-based studies, that utilized mainly objective measures, like reaction time, duration, and accuracy, in the context of behavioral tasks.”

Additionally, there was an error in Table 1 as published. The second-to-final version of Table 1 was included in the original article. The final version of the table appears below.

**Table 1:** Empirical studies in the field of external human–machine interfaces for autonomous vehicle-to-pedestrian communication.

**Studies**	**Stimulus delivery**			**Interface parameters**						**Evaluation procedures**			**Measures**	
	**Physical Prototype**	**Monitor-based**	**VR-based**	**Technology**	**Location**	**Content type**	**Information type**	**Message coding**	**Modality**	**Behavioral task**	**Online survey**	**Questionnaire**	**Objective**	**Subjective**
Hensch et al. (2019)	✓			Display	Roof	Information	Mode, intention	Lights	Visual	Intention identification		Comprehensibility, trust, safety, usefulness		Likert scales, interview
Costa (2017)	✓			Cardboard, speaker	Hood, bumper	Advice		Textual, pictorial, sounds	Visual, auditory	Street-crossing			Frequency	
Mahadevan et al. (2018)	✓			Light strip, display, LEDs, printed hand, mobile phone, speaker	Windshield, hood, roof, street surface, pedestrian's mobile phone	Information	Pedestrian acknowledgment, intention	Lights, speech, vibration,gesture, pictorial	Visual, auditory, haptic	Crossing intention		Effectiveness, confidence		Likert scales, interview
Habibovic (2018)	✓			Light strip	Windshield	Information	Mode, intention	Lights	Visual	Street-crossing		Safety		Likert scales, interview
Clamann et al. (2017)	✓			Display	Radiator grille	Information, advice	Speed	Textual, pictorial	Visual	Street-crossing		Effectiveness	Decision time	Interview
Li et al. (2018)		✓		Display	Windshield, radiator grille, vehicle sides	Advice		Lights	Visual		Situational urgency, crossing intention			Numeric scales, interview
Zhang et al. (2017)		✓		Light strip	Front doors, hood	Information	Intention	Lights	Visual		Intention identification, effectiveness			Interview
Song et al. (2018)		✓		Display	Radiator grille	Advice		Textual, pictorial	Visual		Crossing intention, preference		Reaction time, frequency	Interview
Fridman et al. (2017)		✓		Light strip, display, projection, vehicle lights and signals	Windshield, headlights, fog lights, directional signals, radiator grille, bumper, street surface	Information advice	Intention	Textual, pictorial, lights	Visual		Crossing intention		Error rates, reaction time	
Ackermann et al. (2019)		✓		Light strip, display, projection	Windshield, radiator grille, street surface	Information, advice	Mode	Lights, textual, pictorial	Visual			Comprehensibility, recognizability, ambiguousness, comfort		Numeric scales, interview
Petzoldt et al. (2018)		✓		Light strip	Above license plate	Information	Deceleration	Lights	Visual	Deceleration detection		Usefulness, safety	Error rates, reaction time	Likert scales
Chang et al. (2018)		✓		Light strip, display, projection, rotating vehicle lights	Windshield, radiator grille, street surface, headlights	Information	Intention	Lights, textual, pictorial, anthropomorphism	Visual	Intention identification		Intelligibility	Error rates	Likert scales
Charisi et al. (2017)		✓		Display, light strip, projection, vehicle lights and signals	Windshield, headlights, directional signals, street surface	Information	Intention	Lights, textual, pictorial, anthropomorphism	Visual	Intention identification		Intention identification	Error rates	Interview
de Clercq et al. (2019)			✓	Display, vehicle lights and signals	Radiator grille, frontal brake lights	Information advice	Intention	Textual, lights, pictorial	Visual	Safety-reporting		Safety, preference	Duration	Interview
Hudson et al. (2018)			✓	Display, speaker	Hood	Advice		Textual, pictorial, speech, music	Visual, auditory	Street-crossing		Preference		Interview
Deb et al. (2018)			✓	Display, speaker	Hood	Information advice	Intention	Lights, pictorial, speech, sounds, music	Visual, auditory	Street-crossing		Safety, acceptance	Decision time, duration	Likert scales, interview
Stadler et al. (2019)			✓	Display	Radiator grille	Advice		Lights, textual, pictorial	Visual	Street-crossing		Satisfaction	Error rates, decision time	Numeric scales, interview
Othersen et al. (2018)			✓	Display	Radiator grille	Information	Pedestrian detection, intention	Lights, pictorial	Visual	Street-crossing		Effectiveness, understandability, perceptibility, safety, appeal	Decision time	Interview
Chang et al. (2017)			✓	Rotating vehicle lights	Headlights	Information	Pedestrian acknowledgment, intention	Anthropomorphism	Visual	Crossing intention		Effectiveness, safety	Error rates, reaction time	Likert scales, interview
Böckle et al. (2017)			✓	Light strip, speaker	Vehicle corners	Information	Intention	Lights, sounds	Visual, auditory	Street-crossing		Safety, comfort, effectiveness	Decision time	Likert scales, interview

The authors apologize for these errors and state that they do not change the scientific conclusions of the article in any way. The original article has been updated.
